# Molecular characteristics of mitochondrial DNA and phylogenetic analysis of the hybrid loach of *Misgurnus anguillicaudatus* (female) and *Paramisgurnus dabryanus* ssp. (male)

**DOI:** 10.1080/23802359.2018.1467226

**Published:** 2018-04-28

**Authors:** Guosong Zhang, Xizhai Sun, Guisheng Zhang, Xia Liang, Kejun Cai, Haili Zhang, Daoyu Zhu

**Affiliations:** a School of Agriculture and Bioengineering, Heze University, Heze, China;; b Department of Life Science, Huzhou University, Huzhou, China

**Keywords:** Hybrid loach, *Paramisgurnus*, *Misgurnus*, mitogenome, conservation

## Abstract

The hybrid loach of *Misgurnus anguillicaudatus* (female) and *Paramisgurnus dabryanus* ssp. (male) has the desirable trait of growth performance. Recently, farming scale of this hybrid has been gradually increased in Asia, suggesting a promise of a new variety for loach. In this study, the complete mitochondrial genome of the hybrid loach was obtained by PCR. The genome is 16,646 bp in length, including two ribosomal RNA genes. Thirteen proteins-coding genes, 22 transfer RNA genes, and a non-coding control region, the gene composition and order of were similar to most reported from other vertebrates. Sequence analysis showed that the overall base composition is 29.8% for A, 28.3% for T, 25.6% for C, and 16.3% for G. The phylogenetic tree showed that Misgurnus family got together for one branch, which includes this hybrid loach, and the other loaches had their own branches. Also the mitochondrial genome sequences of the hybrid loach were aligned by BLAST, compared with Cobitidae the sequence similarity could reach >95%, and the similarity to Misgurnus was >99%. The hybrid loach follows the matrilineal inheritance.

Cyprinid loach, *Misgurnus anguillicaudatus* (Cypriniformes; Cobitidae), a small-sized freshwater fish species, is a highly valued, aquaculture-relevant food fish in East Asian countries (Zhang et al. 2014). Taiwanese loach (*Paramisgurnus dabryanus* ssp.) was bred in Formosa and then widely cultivated in China. The hybrid loach of *M. anguillicaudatus* (female) and *P. dabryanus* ssp. (male) has the desirable trait of growth performance. Recently, farming scale of this hybrid has been gradually increased in Asia, suggesting a promise of a new variety for loach. There is no report of the complete genome of this hybrid. Therefore, it is very important to characterize the complete mitogenome of this species, which can be utilized in research on taxonomic resolution, population genetic structure and phylogeography, and phylogenetic relationship (Liu et al. 2016).

In this study, we sequenced the complete mitogenome of the hybrid loach of *M. anguillicaudatus* (female) and *P. dabryanus* ssp. (male) with a GenBank accession number MG938589. The voucher specimen was collected from Zhili Fanyi aquaculture base, north latitude 30°22″ and east longitude 120°25″, Huzhou city, China, which were stored in biology herbarium of Heze University. Its tailfins were preserved in 95% alcohol. All DNAs were extracted using phenol–chloroform extraction methods and stored at –80 °C in Heze University. The mitogenomes were amplified by primers which were initially published (Zeng et al. 2012). The entire mitogenome sequence of the hybrid loach was 16,646 bp in length, consisting of 13 protein-coding genes (PCGs), 2 ribosomal RNA (rRNA) genes, 22 transfer RNA (tRNA) genes, one replication origin (OL) and one control region (D-loop). From the base composition analysis, the percent A + T content was 58.0% (29.8% for A, 28.3% for T, 25.6% for C, and 16.3% for G). Twelve PCGs, 14 tRNA genes and two rRNA genes were located on the heavy strand (H-strand), while one PCG (ND6) and eight tRNA genes (tRNA^Gln^, tRNA^Ala^, tRNA^Asn^, tRNA^Cys^, tRNA^Tyr^, tRNA^Ser^, tRNA^Glu^, and tRNA^Pro^) on the light strand (L strand). Eight PCGs (ND1, COI, COII, ATP8, ATP6, ND4L, ND5, and ND6) were terminated with TAA stop codon and three PCGs (ND2, ND3, and ND4) ended with TAG. On the other hand, remaining two PCGs (COIII and CYTB) ended with the incomplete stop codon represented as a single T. Frame overlapping occurred at three pairs of PCGs. ATP8 and ATP6 overlapped by 10 nucleotides (nt), ND4L and ND4 by 7 nt, and ND5 and ND6 (encoded on opposing stand) by 4 nt. Two rRNA genes, 12S rRNA (953 bp) and 16S rRNA (1679 bp) were located between tRNA^Phe^ and tRNA^Leu^ with a separation by the tRNA^Val^ as seen in other vertebrate mitogenomes.

To determine taxonomic status of the hybrid loach, we performed the phylogenetic relationship of the hybrid loach stock with other natural populations in loach as inferred by entire mitogenome (Dai et al. 2016). The phylogenetic tree showed that Misgurnus family got together for one branch, which includes the hybrid loach, and the other loaches had their own branches (Figure 1). Also the mitochondrial genome sequence of the hybrid loach were aligned by BLAST, compared with Cobitidae the sequence similarity could reach >95%, and the similarity to Misgurnus was >99%. The hybrid loach of *M. anguillicaudatus* (female) and *P. dabryanus* ssp. (male) follows the matrilineal inheritance.

**Figure 1.**
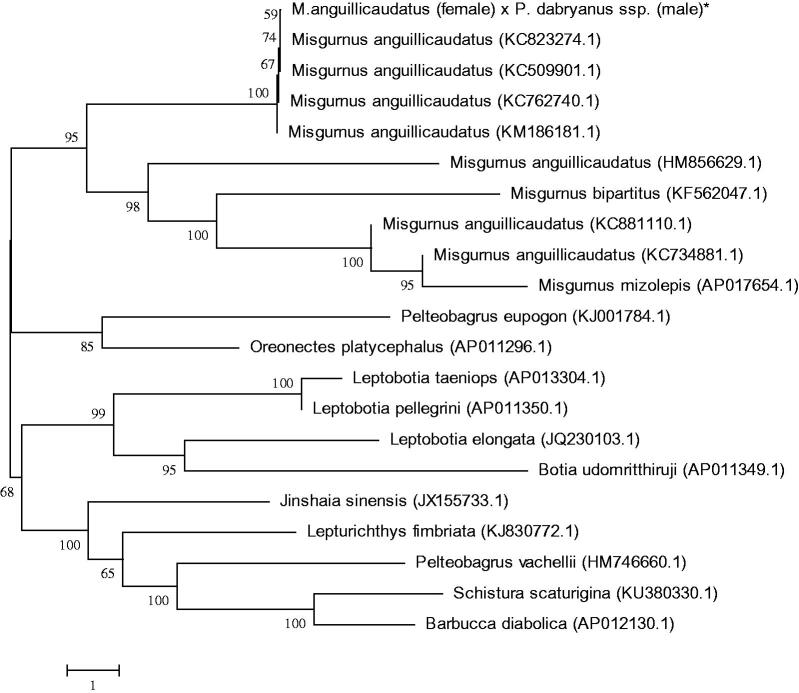
Phylogenetic relationship of the hybrid loach of *M. anguillicaudatus* (female) and *P. dabryanus* ssp. (male) stock with other loach as inferred by entire mitogenome. *The hybrid loach (accession number: MG938589) in the position of the evolutionary tree. Trees were reconstructed using MEGA 7 program (Kumar, Tamura, Nei) with neighbour-joining method. Numbers above branches are bootstrap values by 1000 replicates. The phylogenetic tree showed that the hybrid loach to be one of Misgurnus, and the other loaches had their own branches.
